# Altered phenotypes due to genetic interaction between the mouse phosphoinositide biosynthesis genes *Fig4* and *Pip4k2c*

**DOI:** 10.1093/g3journal/jkad007

**Published:** 2023-01-24

**Authors:** Xu Cao, Guy M Lenk, Miriam H Meisler

**Affiliations:** Department of Human Genetics, University of Michigan, Ann Arbor, MI 48109-5618, USA; Department of Human Genetics, University of Michigan, Ann Arbor, MI 48109-5618, USA; Department of Human Genetics, University of Michigan, Ann Arbor, MI 48109-5618, USA

**Keywords:** modifier, lysosome, FIG4, phosphoinositide, rare disorder

## Abstract

Loss-of-function mutations of *FIG4* are responsible for neurological disorders in human and mouse that result from reduced abundance of the signaling lipid PI(3,5)P_2_. In contrast, loss-of-function mutations of the phosphoinositide kinase *PIP4K2C* result in elevated abundance of PI(3,5)P_2._ These opposing effects on PI(3,5)P_2_ suggested that we might be able to compensate for deficiency of *FIG4* by reducing expression of *PIP4K2C.* To test this hypothesis in a whole animal model, we generated triallelic mice with genotype *Fig 4^−/−^, Pip4k2c^+/−^*; these mice are null for *Fig 4* and haploinsufficient for *Pip4k2c*. The neonatal lethality of *Fig 4* null mice in the C57BL/6J strain background was rescued by reduced expression of *Pip4k2c*. The lysosome enlargement characteristic of *Fig 4* null cells was also reduced by heterozygous loss of *Pip4k2c*. The data demonstrate interaction between these two genes, and suggest that inhibition of the kinase *PIPK4C2* could be a target for treatment of *FIG4* deficiency disorders such as Charcot-Marie-Tooth Type 4J and Yunis-Varón Syndrome.

## Teaser

Cao et al., demonstrate partial rescue of deleterious phenotypes in the mouse null Fig4 mutant by heterozygous loss of function of the kinase Pip4k2c.

## Introduction

Recessively inherited, loss-of-function variants of the phosphoinositide phosphatase *FIG4* are responsible for several rare genetic disorders. Complete loss-of-function of *FIG4* results in Yunis-Varón Syndrome (OMIM 216340), a lethal multisystem disorder affecting development of the skeleton and nervous system (Campeau et al. 2013). Partial loss of *FIG4* function results in the peripheral neuropathy Charcot-Marie-Tooth Type 4J (CMT4J) (OMIM 611228), most often caused by compound heterozygosity for a null allele and the partial loss-of-function variant p.Ile41Thr (Chow et al. 2007; Nicholson et al. 2011). The p.Ile41Thr variant is present at an allele frequency of 0.001 in European populations, and homozygous individuals were recently described (LaFontaine et al. 2021). Other partial loss-of-function variants of *FIG4* result in polymicrogyria with epilepsy (OMIM 612619) (Baulac et al. 2014) and pediatric neurodegeneration with hypomyelination (Lenk et al. 2019a), related to the requirement for PI(3,5)P_2_ during oligodendrocyte maturation (Mironova et al. 2016). Deficiency of the *FIG4* binding partner *VAC14* results in similar neurological disorders (OMIM 617054) (Lenk et al. 2016; de Gusmao et al. 2019).

At the cell level, deficiency of *FIG4* reduces the abundance of the signaling phospholipid PI(3,5)P_2_, leading to defective lysosome function (Chow et al. 2007; Ferguson et al. 2009; Ferguson et al. 2012; Lenk and Meisler, 2014). PI(3,5)P_2_ regulates the activity of lysosomal ion channels and transporters including TRPML1, TPC1, TPC2 and CLC-7 (Dong et al. 2010; She et al. 2018; Patel et al. 2022; Leray et al. 2022). Reduced PI(3,5)P_2_ abundance alters the regulation of lysosomal ion flux and results in osmotic swelling and hyperacidic lysosomes (Chow et al. 2007; Ferguson et al. 2009; Lenk et al. 2019b; Jin et al. 2017; Wilson et al. 2018). Interventions that increase the intracellular levels of PI(3,5)P_2_ could potentially be therapeutic for *FIG4* deficiency disorders.

Chemical inhibition of the phosphoinositide kinase *PIP4K2C* is one intervention that increases intracellular levels of PI(3,5)P_2_ in cultured cells (Al-Ramahi et al. 2017). To determine whether this observation could be reproduced in the whole animal and compensate for loss of *Fig 4*, we carried out crosses between mice with mutations in the two genes. The *Pip4k2c* null mouse exhibits normal growth and viability (Shim et al. 2016). The *Fig 4* null mouse has a lethal phenotype that includes neurodegeneration, diluted pigmentation, and tremor (Chow et al. 2007; Ferguson et al. 2012; Bissig et al. 2019). A partial loss-of-function model of *Fig 4*, which is seen in most patients, is not currently available in the mouse (Lenk et al. 2011). Therefore, to evaluate the effect of reduced *Pip4k2c*, we generated *Fig 4* null mice that are heterozygous for the null allele of *Pip4k2c*.

**Fig. 1.**
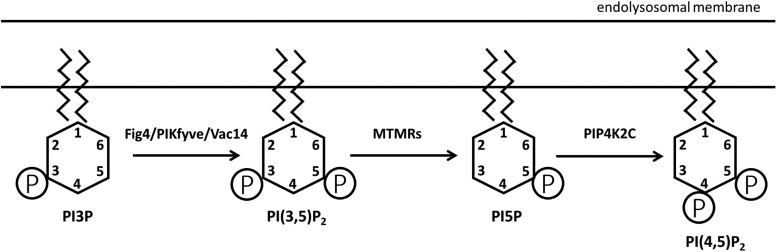
*Fig 4* and *Pip4k2c* in phosphoinositide biosynthesis. Phosphoinositides are localized on the cytoplasmic surface of the endolysosomal compartment. In addition to the enzymes shown here, several additional enzymes catalyze the complex interconversions between the seven naturally occuring phosphoinositides (Hasegawa et al. 2017; Palamiuc et al. 2019). The abundance of the diphosphoinositide PI(3,5)P_2_ is reduced by inactivation of *Fig 4* and increased by inactivation of *Pip4k2c*.

*FIG4* and *PIP4K2C* are components of the phosphoinositide metabolic pathway (Fig. 1). PI(3)P is converted to PI(3,5)P_2_ by a biosynthetic complex that includes the kinase PIKFYVE, the phosphatase FIG4, and the scaffold protein VAC14. The 3-phosphate is removed from PI(3,5)P_2_ by the myotubularins (MTMRs) to generate PI(5)P (Hnia et al. 2012). Subsequent phosphorylation by the kinase PIP4K2C generates PI(4,5)P_2_, an abundant phosphoinositide in cell membranes and synapses (Al-Ramahi et al. 2017).

The FIG4 protein complex is localized on the cytoplasmic surface of endolysosomal vesicles (Jin et al. 2008; Botelho et al. 2008; Lees et al. 2020; Strunk et al. 2020; Rivero-Ríos & Weisman, 2022). PIP4K2C is also localized on cellular vesicles, including the endolysosome (Clarke et al. 2009). Partial loss of PIP4K2C alters the relative levels of the phosphoinositides and elevates PI(3,5)P_2_, resulting in enhanced autophagy and increased turnover of mutant huntingtin protein (Al-Ramahi et al. 2017).

We hypothesized that the increase in PI(3,5)P_2_ resulting from inactivation of *Pip4k2c* might compensate for the deficiency of PI(3,5)P_2_ in *Fig 4* null mice. Consistent with this hypothesis, we observed that heterozygous loss of *Pip4k2c* increased the postnatal survival of *Fig 4* null mice from <1 day to between 1 and 2 weeks and reduced the extensive vacuolization of *Fig 4* null embryonic fibroblasts. This demonstration of genetic interaction suggests that reduction of *Pip4k2c* activity could be therapeutic for genetic disorders of *FIG4*.

## Materials and methods

The spontaneous *Fig 4* null mutation *plt* arose on a mixed genetic background (Chow et al. 2007). The congenic line C57BL/6J.Fig 4^+/−^ was generated by more than 30 generations of backcrossing to wildtype mice of strain C57BL/6J (JAX line 017800). *Pip4k2c* null mice were a product of the mouse knockout mouse project and were generously provided by Dr. Lewis Cantley, Weill Cornell Medical College (Shim et al. 2016). The C57BL/6N strain background of the *Pip4k2c* knockout line was confirmed by genotyping with the miniMUGA panel (Sigmon et al. 2020, Yu et al. 2020)(Neogen Inc. Lincoln, NE). Genotyping of *Pip4k2c* mice was carried out as previously described (Shim et al. 2016). The *Fig 4* mutation was genotyped by PCR with the forward primer 5′-CTTCT TTGGT GACAG GAAGA TAGA-3′ and two reverse primers. Reverse primer 5′-AGACC ACTGA AGGAT GTAGA TGTG-3′ amplifies a 200 bp fragment from the wildtype allele, and reverse primer 5′-GGAGC TAAGG CAATT TCATA CTG-3′ amplifies a 400 bp fragment from the *plt* mutant allele. Mouse embryonic fibroblasts (MEFs) were prepared 13 days after detection of a plug (day E13.5).

## Results

### Neonatal lethality of *Fig 4^−/−^* mice

*Fig 4^−/−^* null mice were generated by timed matings between congenic C57BL/6J.*Fig 4^+/−^* heterozygotes. *Fig 4^−/−^* embryos were detected at the predicted Mendelian frequency of 25% during prenatal development (15/65 total) (Table 1). At postnatal day 3, only 1 *Fig 4^−/−^* mouse was identified among 87 live births. We previously showed that *Fig 4^−/−^* homozygotes survive for 3–5 weeks on the C3HeB/FeJ or mixed strain background (Chow et al. 2007; Lenk and Meisler, 2014; Presa et al. 2015). In the current study, the congenic C57BL/6J.*Fig 4^+/−^* mice were used.

**Table 1.** Neonatal lethality of *fig 4* null mice on strain C57BL/6j. Timed matings between *Fig 4^+/−^* mice were carried out to determine the age at loss for *Fig 4* null mice on the congenic C57BL/6J strain background. The probability (*P*-value) for the predicted Mendelian inheritance of 25% was calculated with the chi-squared test.

Age	*Fig 4^−/−^*	Total	% *Fig 4^−/−^*	*P*-value
E14.5	4	21	19%	0.7
E16.5	4	15	27%	1.0
E17.5	2	10	20%	0.6
E18.5	5	19	26%	1.0
P3	1	87	1%	<0.0001

### Rescue of Fig 4^−/−^ mice by null heterozygosity for Pip4k2c^+/−^

**Fig. 2.**
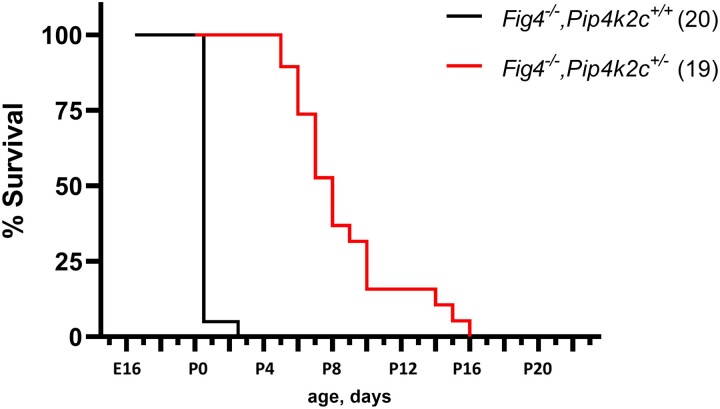
Effect of *Pip4k2c* on survival of *fig 4* null mice*. Fig 4* null mice with wildtype *Pip4k2c* are viable through prenatal life but do not survive after birth. (see also Table 1). Heterozygosity for the null allele of *Pip4k2c* rescues neonatal lethality, with mean survival of 8.2 days and maximum survival of 16 days.

*Pip4k2c^−/−^* null males were crossed with *Fig 4^+/−^* females to produce double heterozygous offspring with genotype *Fig 4^+/−^, Pip4k2c^+/−^* (Fig. 2). A subsequent intercross between double heterozygotes generated 304 F2 mice. Mice were genotyped on P1. The yield of *Fig 4* null mice in the F2 was 0, compared with the prediction of 1/16 or 19/304. The lack of *Fig 4* null mice in this F2 is consistent with the postnatal yield of only 1/87 *Fig 4* null mice in the cross between *Fig 4* heterozygotes shown in Table 1. The combined yield of *Fig 4* null mice from the two crosses was 1 mouse compared with the combined prediction of 41 null mice (19 from the F2 and 22 from Table 1).

The addition of the *Pip4k2c* null allele to the lethal *Fig 4* null genotype resulted in 50% restoration of viability. The yield of F2 mice with genotype *Fig 4^−/−^, PIP4k2c^+/−^* was 22, compared with the prediction of 1/8 or 38/304. Comparing the two outcome of both crosses, the yield of *Fig 4* null mice was increased from 1/41 to 22/38 by combination with the *Pip4k2c* null allele in the F2; this represents a significant increase in viability (*P* < 0.0001, Fisher's exact test).

### Lifespan is extended in *Fig 4^−/−^, Pip4k2c^+/−^* mice

The average survival time of *Fig 4^−/−^, Pip4k2c^+/−^* mice from the F2 cross was 8.6 ± 3.2 days (*n* = 22), with maximum survival of 16 days (Fig. 2). In contrast, none of the Fig 4^−/−^ mice from the F2 survived beyond P0. Heterozygosity for a null allele of *Pip4k2c* thus increases the postnatal viability of *Fig 4* null mice (Fig. 2).

### *Pip4k2c^+/−^* genotype also rescues enlarged lysosomes in mouse embryonic fibroblasts

**Fig. 3.**
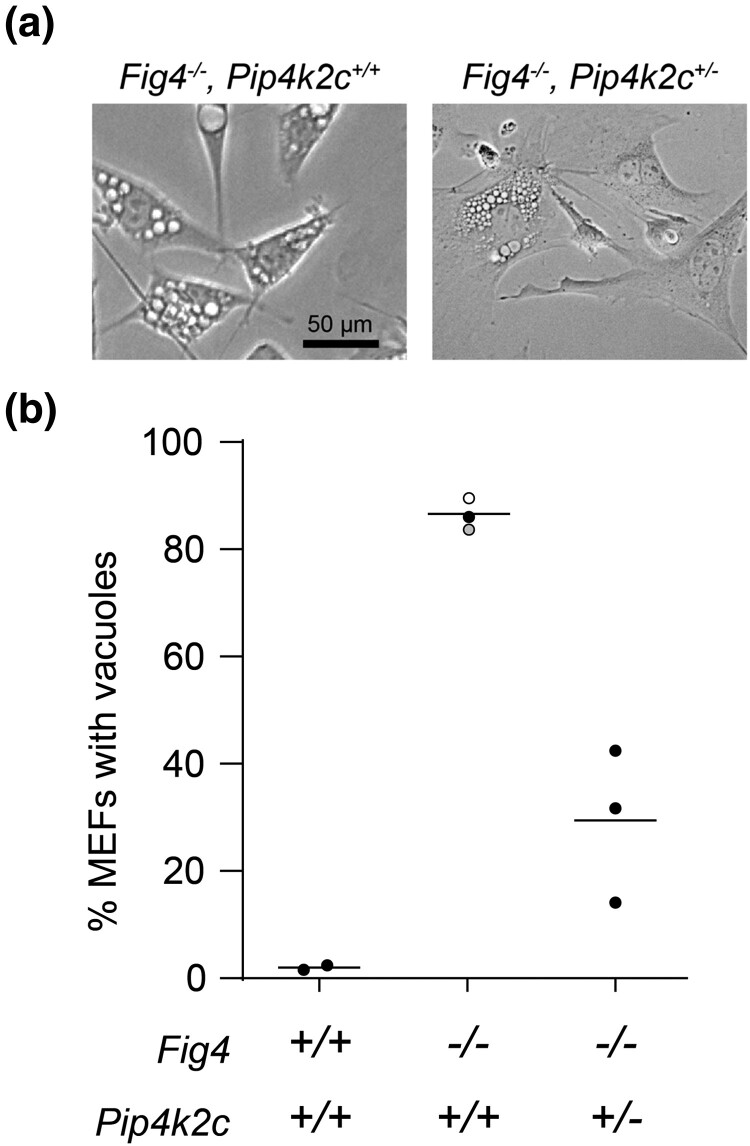
Heterozygous loss of *Pip4k2c* reduces vacuolization of *fig 4* null MEFs. Mouse embryonic fibroblasts were isolated at day E13.5 and examined by phase contrast microscopy. (a) Each symbol in (b) represents the percent of vacuolated MEFs from one mouse, ≥ 200 cells per mouse. Solid symbols, this study; open symbol, Campeau et al. 2013; gray symbol, Lenk et al. 2016.

To investigate the mechanism of rescued viability, we examined the appearance of lysosomes from mouse embryonic fibroblasts (MEFs) cultured at E13.5 from F2 mice. Most of the *Fig 4* null cultured cells exhibit extensive enlargement of LAMP-positive, acidic vacuoles (Fig. 3a) (Ferguson et al. 2009; Lenk et al. 2019b). In contrast, many of the *Fig 4^−/−^, PIP4k2c^+/−^* fibroblasts lacked vacuoles (Fig. 3a). Quantitatively, 82% of MEFs from the *Fig 4^−/−^* mice exhibited extensive intracellular vacuolization while only 29% of MEFs from *Fig 4^−/−^, Pip4k2c^+/−^* mice contained enlarged vacuoles (Fig. 3b). The increased viability of the *Fig 4^−/−^, PIP4k2c^+/−^* mice is thus correlated with correction of the lysosome phenotype. The data demonstrate an effect of *Pip4k2c* haploinsufficiency on the lysosomal function of PI(3,5)P_2_ and provide a cellular basis for the increased viability of *Fig 4^−/−^, Pip4k2c^+/−^* mice.

## Discussion

The experiments described here demonstrate interaction between *Fig 4* and *Pip4k2c* in regulation of lysosome function. The kinase PIP4K2C phosphorylates PI5P to generate PI(4,5)P_2_ (Fig. 1). Inhibition of PIP4K2C alters the relative proportions of these phosphoinositides (Al-Ramahi et al. 2017). The resulting elevation of PI(3,5)P_2_ may reflect the buildup of precursors due to the downstream block in PIP4K2C enzymatic activity. Alternatively, PIK4K2C is known to inhibit the kinase PIP5K by direct protein interaction (Wang et al. 2019). If PIP4K2C also inhibits the kinase PIKfyve, then relief of that inhibition in heterozygous null *PIP4K2C* cells could directly increase production of PI(3,5)P_2_. Regardless of the underlying mechanism, reduction of PIP4K2C provides an intervention for in vivo elevation of PI(3,5)P_2_.

Knockout of the phosphatase MTMR2 (Fig. 1) increases the concentration of PI(3,5)P2 in fibroblasts from Fig 4^+/+^ mice, but does not increase the concentration in fibroblasts from Fig^−/−^ mice (Vaccari et al. 2014). The basis for the different effects in wildtype and mutant cells is not known. *In vivo*, the phenotype of Fig 4^−/−^ mice was exacerbated by reduction of MTMR2 in the earlier work, in contrast to the improvement after reduction of the kinase PIP4K2C reported here. The different effects of reducing MTMR2 and PIP4K2C in fibroblasts and in mice may reflect their roles in metabolism of other substrates in this complex pathway.

In *Fig 4* null fibroblasts, the level of PI(3,5)P_2_ is reduced to 50% of that of wildtype cells (Chow et al. 2007). The low level of PI(3,5)P_2_ results in lysosomal vacuolization and neonatal lethality. We have demonstrated that vacuolization and neonatal lethality can be corrected by 50% reduction of *Pip4k2c via* heterozygous knockout in *Fig 4* null mice. This observation introduces a new paradigm for treatment of *FIG4* deficiency.

Although striking, the beneficial effects in *Fig 4^−/−^, Pip4k2c^+/−^* mice are transient and maximal survival was observed at 2 weeks postnatal. This temporal limitation may be a consequence of the normal developmental increase in *Pip4k2c* expression that occurs between 1 and 2 weeks postnatal (Clarke et al. 2009). The developmental increase could elevate *Pip4k2c* level in the heterozygous null mouse beyond a critical threshold and eliminate the clearly beneficial effects seen during the first week of postnatal life. Alternatively, in vivo correction in neurons may be less efficient than observed in cultured cells.

The F2 cross that we carried out also generated double null mice with the genotype.

*Fig 4^−/−^, Pip4k2c^−/−^*. However, the very low yield of double null mice and their variable phenotype made it impossible to include them in the study.

The active site of the kinase *Pip4k2c* is a “druggable target” that has been investigated as a modifier of protein turnover and metastasis. The PIP4K2C inhibitor phenazopyridine is a widely used over-the-counter drug for treatment of urinary tract pain (Preynat-Seauve et al. 2021). The selective inhibitor NCT-504 alters the proportions of phosphoinositides in cultured cells and enhances autophagy; this inhibitor was identified in a screen for enhanced degradation of mutant huntingtin (Al-Ramahi et al. 2017). The covalent *Pip4k2c* inhibitor THZ-P1-2 causes defects in autophagy similar to those caused by inactivation of the gene (Sivakumaren et al. 2020). Our studies suggest that the new generation of pharmacological inhibitors of PIP4K2C could be applied to the PI(3,5)P_2_ deficiency disorders caused by mutations of *FIG4* and *VAC14*.

We observed positive effects of reduced *Pip4k2c* in mice with a severe disorder due to complete loss of *Fig 4*. Since most patients have only partial loss of *FIG4*, the effectiveness might be greater and longer-lasting in the human disorders. Long-term rescue of the *Fig 4* null mice was recently reported by gene replacement using viral delivery of the *Fig 4* cDNA (Presa et al. 2021). The gene replacement did not completely restore normal expression, and combination of viral therapy with small molecule inhibitors of *Pip4k2c* might prove useful. Until gene replacement becomes available in the clinic, targeting of *Pip4k2c* offers an alternative for amelioration of the severe effects of *Fig 4* deficiency.

## Data Availability

Strains and plasmids are available upon request. The authors affirm that all data necessary for confirming the conclusions of the article are present within the article, figures, and tables.
